# Response Surface Optimization for Water-Assisted Extraction of Two Saponins from *Paris polyphylla* var. *yunnanensis* Leaves

**DOI:** 10.3390/molecules29071652

**Published:** 2024-04-06

**Authors:** Yutian Jin, Qing Qiao, Linmei Dong, Mokun Cao, Ping Li, Aizhong Liu, Rui Sun

**Affiliations:** Key Laboratory for Forest Resources Conservation and Utilization in the Southwest Mountains of China, Ministry of Education, Southwest Forestry University, Kunming 650224, China

**Keywords:** *Paris polyphylla* var. *yunnanensis*, saponins, extraction process, Box–Behnken response surface methodology, water-assisted extraction

## Abstract

The process of extracting polyphyllin II and polyphyllin VII by water-assisted extraction was established and optimized in this study. Response surface methodology was used to establish a prediction model to optimize the extraction conditions. Based on the one-way test, the Box–Behnken design with three factors and three levels was used for the experimental program, and the composition analysis was carried out by high-performance liquid chromatography (HPLC). The optimal extraction conditions for polyphyllin II and polyphyllin VII were as follows: extraction time of 57 and 21 min, extraction temperature of 36 and 32 °C, solid-to-liquid ratio of 1:10 and 1:5 g/mL, respectively, and the yields of polyphyllin II and polyphyllin VII were 1.895 and 5.010%, which was similar to the predicted value of 1.835 and 4.979%. The results of the ANOVA showed that the model fit was good, and the Box–Behnken response surface method could optimize the water-assisted extraction of saponins from the leaves of *Paris polyphylla* var. *yunnanensis*. This study provides a theoretical basis for the application of polyphyllin II and polyphyllin VII in pharmaceutical production.

## 1. Introduction

*Paris polyphylla* Smith var. *yunnanensis* (Franch.) Hand.-Mazz.(*P. polyphylla* var. *yunnanensis*) is a perennial herb of the genus Paris in the family Trilliaceae. Steroidal saponins, the main active ingredients in *P. polyphylla* var. *yunnanensis*, have hemostatic, immunomodulatory, and antitumor effects [1,2,3]. In recent years, the antitumor research of steroidal saponin extract from *P. polyphylla* var. *yunnanensis* has become hot, which can inhibit the growth of malignant tumors, such as lung cancer, colon cancer, liver cancer, bladder cancer, and so on [4,5,6,7]. Polyphyllin II was found to promote Ros-mediated apoptosis of Bax/Cyt-c cells, leading to the inhibition of glioma cell growth and invasion [8]. The anticancer activity of polyphyllin VII could induce autophagic cell death through the activation of the JNK pathway and inhibition of the PI3K/AKT/mTOR pathway in HepG2 cells [9].

**Figure 1:**
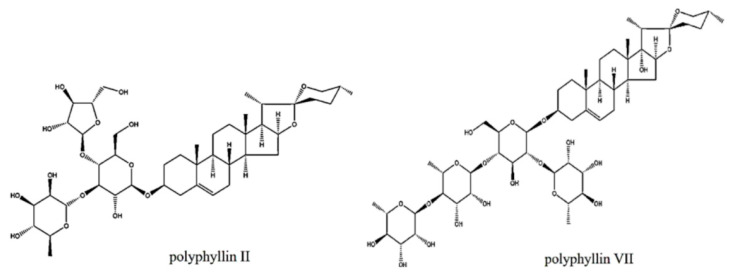
Chemical structures of two saponins.

Paridis Rhizoma is a type of traditional Chinese medicine that is made from the dried rhizomes of *P. polyphylla* var. *yunnanensis* [10]. It is widely used in various proprietary Chinese medicines such as Antiviral Granules, Bolnin Capsules, and Ji De sheng Snake Pill [11]. The stable and precise therapeutic effects of *P. polyphylla* var. *yunnanensis* and the discovery of new functions have led to an increase in the demand for it year by year, resulting in the decline of wild populations [12]. In addition to expanding the area of artificial cultivation, we should also improve its utilization to reduce waste. However, the above-ground parts (leaves and stems) of *P. polyphylla* var. *yunnanensis* have always been considered to have no medicinal value and are usually discarded as wastes without being utilized. Research has shown that the saponins contained in the leaves of *P. polyphylla* var. *yunnanensis* are similar to those in the rhizomes, such as polyphyllin II and polyphyllin VIII (Figure 1) [13], and it was even found that the synthesis of polyphyllin VII was mainly in the leaves [14]. Therefore, it is the focus of this study to find an economical and practical extraction method to improve the extraction efficiency of the medicinal components contained in the leaves of *P. polyphylla* var. *yunnanensis*.

The common methods of plant saponins extraction are enzyme-assisted extraction [15], microwave-assisted extraction [16], ultrasonic-assisted extraction [17], and so on. Research has found that water could be a potential green solvent [18,19]. The properties of water can be regulated by changing the temperature and pressure [20], so studies have found that hot water can disrupt the structure of some cell walls, which helps to dissolve substances out of plant cells [21,22]. However, a study on the extraction of polyphyllin II and polyphyllin VII from *P. polyphylla* var. *yunnanensis* using water-assisted extraction has not yet been reported. After the preliminary investigation, it was found that although the enzyme-assisted extraction of saponins from *P. polyphylla* var. *yunnanensis* had a higher yield, the effect of water-assisted extraction was close to that of enzyme-assisted extraction, and both had a higher yield of saponins than that of conventional direct ethanol extraction.

Response surface methodology (RSM) is an optimization tool that can identify the interrelationships between variables. Compared to traditional full factorial design, the number of trials can be greatly reduced while still allowing for the testing of all variables in a shorter period. This method is particularly cost-effective for high-cost samples and provides a more economical alternative to the traditional one-factor optimization method [23]. The response surface methodology includes three main experimental design schemes: Box–Behnken design (BBD) [24], Central Composite Design (CCD) [25], and Doehlert Design (DD) [26]. BBD is a widely used experimental design method that aims to determine the optimal variables for the best response/output in multifactorial optimization experiments. DD derived from spherical domains has some advantage in some scenarios, but it does not have the classical properties of response surface designs [26]. BBD is more efficient compared to CCD, and using BBD can help avoid unsatisfactory results under extreme conditions [24]. In recent years, it has been frequently used in optimization experiments of the extraction process, and the results have shown that it is able to optimize the extraction conditions better and has good predictability of the conditions [27,28]. Optimizing polyphyllin II and polyphyllin VII extraction conditions from *P. polyphylla* var. *yunnanensis* leaves using a response surface approach has not been studied before. Therefore, the results of this study using a water-assisted extraction method are important.

The objective of this study was to establish a new and improved water-assisted extraction process for polyphyllin II and polyphyllin VII based on Box–Behnken design using *P. polyphylla* var. *yunnanensis* leaves as the research object. It provides the theoretical basis for the application of polyphyllin II and polyphyllin VII in pharmaceutical production.

## 2. Results and Discussion

### 2.1. Pre-Experimentation

Pre-experiments were conducted to investigate the effects of ethanol extraction (control group), water-assisted extraction, and enzyme-assisted extraction on the yields of polyphyllin II and polyphyllin VII, respectively.

**Figure 2:**
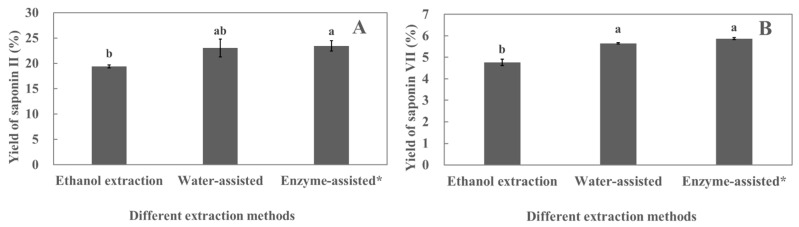
Effect of different extraction methods on the yields of polyphyllin II (**A**) and polyphyllin VII (**B**). * indicates that the enzyme used pectinase, and the concentration of the pectinase solution is 0.5 mg/mL. Different lower cases above the columns indicate significant differences between saponin yields (*p* < 0.05). Each value is the mean of triplicate measure, *n* = 3.

As shown in Figure 2, the yields of polyphyllin II and polyphyllin VII extracted by water-assisted and enzyme-assisted extraction methods were significantly higher than those extracted by the conventional ethanol extraction method. The yield of polyphyllin VII extracted by the water-assisted extraction method was not significantly different from that extracted by the enzyme-assisted extraction method (Figure 2B). It was found that some pectic polysaccharides can be extracted from the cell wall in hot water [21,22], leading to the weakening of cell wall integrity. Saponins can be released from cells, thereby increasing the yield of saponins. Furthermore, in the process of water-assisted extraction, water can activate the plant’s enzymes, such as pectinase and cellulase, under specific conditions, causing hydrolysis reactions that break down the cell wall, making it easier for the release of active ingredients. Therefore, in this study, the extraction process of the water-assisted extraction of polyphyllin II and polyphyllin VII from the leaves of *P. polyphylla* var. *yunnanensis* will be investigated.

### 2.2. Single-Factor Experiment

**Figure 3:**
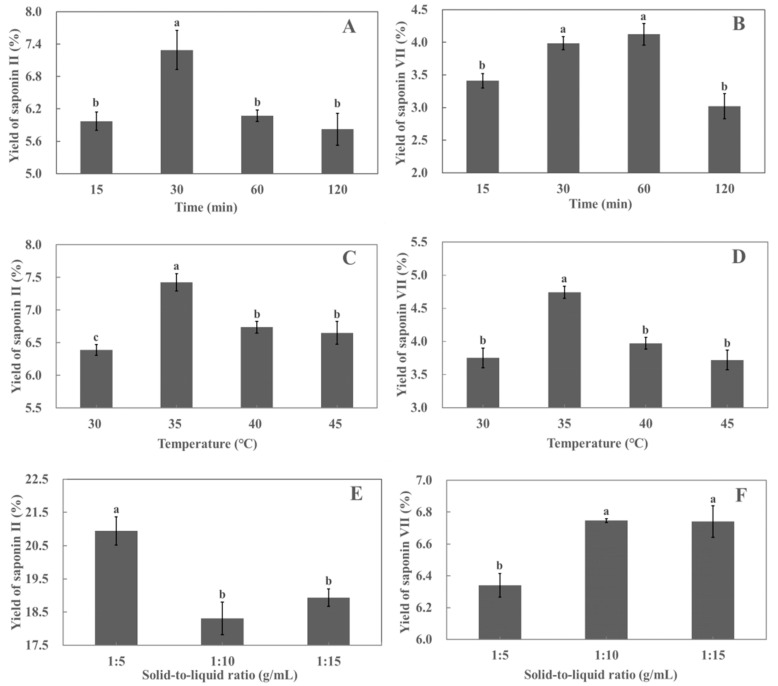
Effect of different extraction periods on the yields of polyphyllin II (**A**) and polyphyllin VII (**B**); effect of different extraction temperatures on the yields of polyphyllin II (**C**) and polyphyllin VII (**D**); effect of different solid-to-liquid ratios on the yields of polyphyllin II (**E**) and polyphyllin VII (**F**). Different lower cases above the line graph indicate significant differences between saponin yields (*p* < 0.05). Each value is the mean of triplicate measure, *n* = 3.

The effect of different periods (15, 30, 60, and 120 min) on the yield of polyphyllin II and polyphyllin VII from the leaves of *P. polyphylla* var. *yunnanensis* was investigated. As shown in Figure 3A, in the extraction period of 30 min, the polyphyllin II yield increased with the extension of time, and the yield of polyphyllin II reached the highest value (6.04%) at 30 min, following which the yield of polyphyllin II started decreasing. As shown in Figure 3B, when the extraction period was within 60 min, the rate of polyphyllin VII increased with time, and the yield of polyphyllin VII reached a maximum (4.12%) at 60 min, following which the yield of polyphyllin VII decreased with time. The increase in yield may be due to the mechanical effect of ultrasound, which breaks down the cell wall to dissolve the saponins [29]. In addition, certain plant enzymes such as pectinase and cellulase may activate, causing cell wall rupture and promoting saponins’ release. As the extraction period increases, more saponin compounds dissolve into the solvent until the solvent becomes saturated [30]. Meanwhile, long ultrasonic extraction time will destroy the saponin compounds [31], so the yields of polyphyllin II and polyphyllin VII decrease. In summary, the yields of polyphyllin VII were not significantly different at 30 and 60 min. Moreover, since polyphyllin II and polyphyllin VII were derived from the same sample, the extraction period for the RSM study was set to a complete study range of 15–120 min.

The effects of different temperatures (30, 35, 40, and 45 °C) on the yields of polyphyllin II and polyphyllin VII are shown in Figure 3C,D. When the extraction temperature was increased from 30 °C to 35 °C, the yields of polyphyllin II and polyphyllin VII increased with the increased temperature. The yields of polyphyllin II and polyphyllin VII reached maximums at 35 °C. This may be due to the acceleration of molecular motion with increasing temperature, which increases the solubility of saponins [32]. As the temperature exceeded 35 °C, the yields of polyphyllin II and polyphyllin VII decreased. It has been proven through previous studies that most natural compounds are unable to inherit high temperatures [33,34]. Considering that there was no significant difference between the yields of polyphyllin II and polyphyllin VII at 40 °C and 45 °C, extraction temperatures of 30–40 °C were chosen for the RSM study.

The effects of different solid-to-liquid ratios (1:5, 1:10, and 1:15 mg/mL) on the yields of polyphyllin II and polyphyllin VII are shown in Figure 3E,F. The maximum yield of polyphyllin II was obtained at a solid-to-liquid ratio of 1:5 g/mL. However, increasing the solid-to-liquid ratio above 1:5 g/mL resulted in a decrease in the yield of polyphyllin II. On the contrary, the lowest yield of polyphyllin VII was obtained at a solid-to-liquid ratio of 1:5 g/mL. However, increasing the solid-to-liquid ratio above 1:5 g/mL increased the yield of polyphyllin VII. It is indicated that different saponin compounds had different solubilities at the same solid-to-liquid ratio. Further process optimization research is needed for each compound. Therefore, the solid-to-liquid ratio of 1:5–1:15 g/mL was chosen for the RSM study.

### 2.3. Establishment of the Regression Equation and ANOVA

Table 1 shows the results of the response surface tests for polyphyllin II and polyphyllin VII. To analyze the effect of independent variables on polyphyllin II yield, a quadratic multiple regression model equation was fitted by using the experimental data of polyphyllin II:

Y = 1.85 − 0.0048A + 0.0112B + 0.0083C − 0.0279AB + 0.0270AC − 0.0261BC − 0.0268A^2^ − 0.0473B^2^ − 0.0342C^2^

Based on the results of the analysis of variance (ANOVA) shown in Table 2, it was apparent that the regression model was extremely significant (*p* < 0.0001), with an F-value of 37.41; the lack of fit was not significant (*p* > 0.05), with an F-value of 2.59. The correlation coefficient (R^2^) of the model was 0.9796, and the adjusted coefficient of determination (R^2^_Adj_) was 0.9535, which indicated that the regression model was well fitted. Meanwhile, the low coefficient of variation (CV) value (0.52%) means that the actual value has high accuracy and good reliability. The significance test of the model showed that the effect of the square term B^2^ reached an extremely significant level (*p* < 0.001). The effects of the square terms A^2^, C^2^ and the interaction terms AB, AC, and BC reached a highly significant level (*p* < 0.01). The effects of the primary terms (B and C) and the interaction term (CD) reached a significant level (*p* < 0.05). The effects of each factor on the yield of polyphyllin II were in the following order: B > C > A, i.e., extraction temperature > solid-to-liquid ratio > extraction period.

The experiment data of polyphyllin VII were used to fit a quadratic multiple regression model equation:

Y = 4.62 − 0.3073A − 0.2705B − 0.2007C − 0.0623AB − 0.1071AC + 0.0250BC − 0.0883A^2^ − 0.1744B^2^ − 0.0298C^2^

The ANOVA of the regression equations in Table 3 showed that the regression model was extremely significant (*p* < 0.0001), with an F-value of 29.09, and the lack of fit was not significant (*p* > 0.05), with an F-value of 1.62. The R^2^ of the model was 0.9740, and the R^2^_Adj_ was 0.9405, which indicated that the regression model was well fitted. Meanwhile, the low CV value (1.90%) means that the actual value has high accuracy and good reliability. The significance test of the model showed that the effects of the primary terms A and B were extremely significant (*p* < 0.001). The effects of the primary term C and the squared term B^2^ were highly significant (*p* < 0.01). The effect of the interaction term AC was significant (*p* < 0.05). The effects of each factor on the yield of polyphyllin VII were in the following order: A > B > C, i.e., extraction period > extraction temperature > solid-to-liquid ratio.

### 2.4. Optimization of the Extraction Conditions and Verification of the Mode

**Figure 4:**
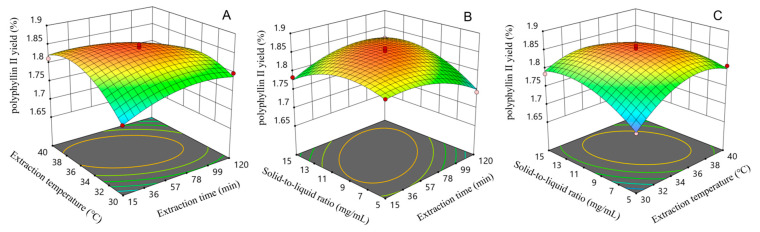
Response surface curve plots of the interactive effects of different factors on polyphyllin II yield: (**A**) interaction between extraction time and extraction temperature; (**B**) interaction between solid-to-liquid ratio and extraction time; (**C**) interaction between solid-to-liquid ratio and extraction temperature.

**Figure 5:**
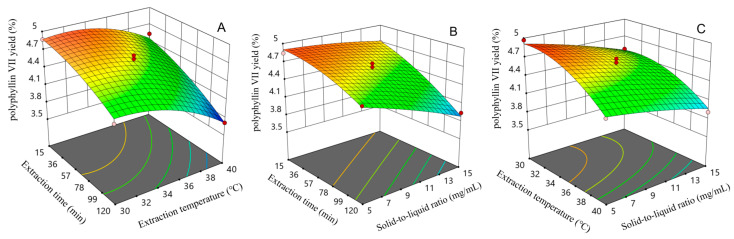
Response surface curve plots of the interactive effects of different factors on polyphyllin VII yield: (**A**) interaction between extraction time and extraction temperature; (**B**) interaction between solid-to-liquid ratio and extraction time; (**C**) interaction between solid-to-liquid ratio and extraction temperature.

The regression equations were analyzed with Design Expert software (version 13.0) to obtain the three-dimensional response surface curve plots of the interaction effects of the factors on the yields of polyphyllin II and polyphyllin VII. The results are shown in Figure 4 and Figure 5. When analyzing three-dimensional response surface curve plots, the steepness of the curves and the elliptical shape of the contours indicate that there are significant interactions between variables that affect the response values. When analyzing three-dimensional response surface curve plots, the steepness of the curves and the elliptical shape of the contours indicate that there are significant interactions between variables that affect the response values. On the other hand, if the curves are gentle and the contours are rounded, it suggests that the effect of these variables is not significant [35,36,37].

By comparing the two-factor interactions on the yield of the polyphyllin II, it can be seen that the three interactions of extraction period and extraction temperature (Figure 4A), extraction temperature and solid-to-liquid ratio (Figure 4B), and extraction period and solid-to-liquid ratio (Figure 4C) have steeper response surface curves, so they have a significant effect on the yield of obtaining polyphyllin II, which is consistent with the results of the ANOVA. As shown in Figure 4A, the polyphyllin II yields were maximized at certain extraction temperatures and extraction solid-to-liquid ratio under the central conditions of extraction time. Figure 4B demonstrates that when the extraction temperature was kept constant and the solid-to-liquid ratio kept changing, the yield of polyphyllin II tended to increase and then decrease with the extension of extraction time. Figure 4C shows the significant effect of the solid-to-liquid ratio on the yield of polyphyllin II for a given extraction time and extraction temperature. According to the mathematical model obtained by RSM, the optimal parameters for the extraction of polyphyllin II were extraction time of 56.972 min, extraction temperature of 35.928 °C, and solid-to-liquid ratio of 1:9.861 g/mL.

Upon comparing the effect of two-factor interaction on the yield of polyphyllin VII, it was observed that the response surface curve plot of the interaction between the extraction period and extraction temperature was steeper. This indicated that the interaction had a significant effect on the yield of polyphyllin VII, which was consistent with the results of the ANOVA. According to Figure 5A, the yield of polyphyllin VII was found to be higher when the extraction period was less than 36 min, and the extraction temperature was below 36 °C. Similarly, Figure 5B suggests that the yield of polyphyllin VII was higher when the extraction period was less than 57 min and the solid-to-liquid ratio was lower than 1:11 g/mL. As shown in Figure 5C, it was found that the yield of polyphyllin VII was higher when the extraction temperature was below 34 °C and the solid-to-liquid ratio was less than 1:7 g/mL. Based on the mathematical model obtained from RSM, the optimal extraction conditions for polyphyllin VII were extraction time of 20.681 min, extraction temperature of 31.560 °C, and solid-to-liquid ratio of 1:5 g/mL.

### 2.5. Response Surface Methodology Extracted Variables Optimization and Validation

A three-factor, three-level Box–Behnken design was used to optimize the independent variables of extraction period, extraction temperature, and solid-to-liquid ratio. The optimal parameters for the extraction of polyphyllin II and polyphyllin VII were obtained as follows: extraction period of 56.972 and 20.681 min, extraction temperatures of 35.928 and 31.560 °C, and solid-to-liquid ratio of 1:9.861 and 1:5 g/mL, respectively. Considering the practical feasibility, the above optimal process conditions were adjusted to extraction periods of 57 and 21 min, extraction temperatures of 36 and 32 °C, and solid-to-liquid ratio of 1:10 and 1:5 g/mL, respectively. As shown in Table 4, three replicate experiments were carried out under the corresponding conditions, and the yields of polyphyllin II and polyphyllin VII were 1.895 and 5.010%, which were similar to the predicted values of 1.835 and 4.979%, respectively. Moreover, the RSDs of the three replicates of the experiments for the two saponins were 2.300 and 1.270%, respectively, indicating good results. The results showed that the model was feasible for parameter optimization of the extraction process of polyphyllin II and polyphyllin VII.

## 3. Materials and Methods

### 3.1. Materials and Reagents

The *P. polyphylla* var. *yunnanensis* plant was purchased from Yunnan Kunming Jiange Herbal Medicine Plantation Co. (Kunming, China). The 98% purity standards of polyphyllin II and polyphyllin VII were purchased from Shanghai Dande Biotechnology Co. (Shanghai, China). Analytical-grade ethanol was purchased from Guangdong Guanghua Technology Co. (Shantou, China). Chromatographic acetonitrile was purchased from Beijing Myriad Technology Co. Ultrapure water from ELGA (PP010XXM1, Birmingham, UK) was used for all the experiments. Pectinase was purchased from Tokyo Kasei Kogyo Co. (Tokyo, Japan). Liquid nitrogen was purchased from Yunnan Ruida Dry Ice Manufacturing Co. (Kunming, China). All the prepared high-performance liquid chromatography (HPLC) solvents were filtered through a microporous filter membrane with a pore size of 0.22 µm before use.

### 3.2. HPLC Analysis of Polyphyllin II and Polyphyllin VII

Polyphyllin II and polyphyllin VII were analyzed on an Agilent 1200 liquid chromatography system (Agilent Technologies, Inc., Santa Clara, CA, USA) equipped with a quaternary pump (G1311A, GER), an autosampler (G1329A, GER), a DAD UV detector (G1315D, GER), and an EC-C18 column (250 mm × 4.6 mm id; 4 µm). The gradient elution method (Table 5) was used with water and acetonitrile as mobile phases at a flow rate of 1.0 mL/min. The column temperature was set at 30 °C, the detection wavelength was set at 203 nm, the injection volume was 10 μL, and the analytical time for a single sample was 25 min.

**Figure 6:**
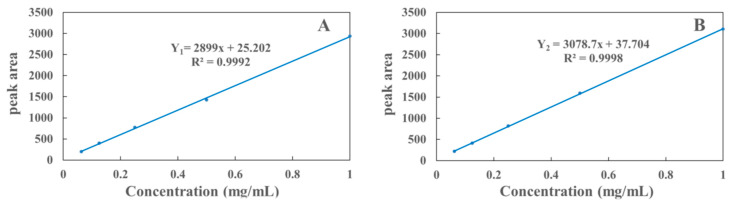
Standard curves of polyphyllin II (**A**) and polyphyllin VII (**B**).

The quantitative analysis in this experiment was performed by the external standard method, which showed good linearity in the range of 0.016–1 mg/mL. The standard curve was plotted with the concentration of polyphyllin II and polyphyllin VII as the X-axis of the coordinate system and the peak area as the Y-axis of the coordinate system. As shown in Figure 6, the regression equation of the standard curve is as follows:

Y_1_ = 2899X + 25.202 (R^2^ = 0.9992)

Y_2_ = 3078.7X + 37.704 (R^2^ = 0.9998)

where Y_1_ is polyphyllin II, Y_2_ is polyphyllin VII, and X is the independent variable.

### 3.3. Pre-Experimentation

**Figure 7:**
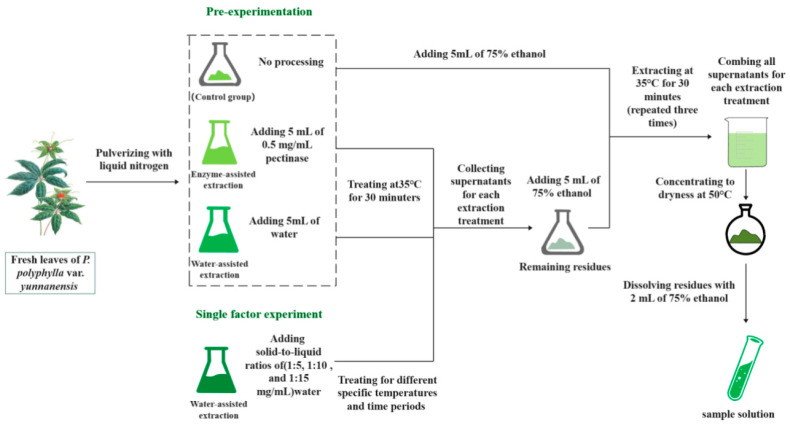
Experimental scheme for extraction of polyphyllin II and polyphyllin VII.

The fresh leaves of *P. polyphylla* var. *yunnanensis* were ground into powder using liquid nitrogen. A total of 1 g of leaf powder was treated with 5 mL of 0.5 mg/mL pectinase and 5 mL of pure water in an ultrasonic cleaner (K5210HP: Shanghai KD Ultrasonic Instrument Co., Ltd., Shanghai, China) at 35 °C for 30 min, respectively, and then extracted with 5 mL of 75% ethanol in an ultrasonic cleaner at 35 °C for 30 min, and the process was repeated three again. In total, 1 g of leaf powder in the control group was extracted directly with 5 mL of 75% ethanol three times in an ultrasonic cleaner with no treatment. In the extraction process of three experimental groups (control group, enzyme-assisted extraction group, and water-assisted extraction group), the ultrasonic cleaner was used to better damage the cell wall and facilitate the sufficient extraction of active ingredients. The filtered solutions were combined and concentrated to dryness at 50 °C in a rotary evaporator device (Model N-1300 rotary evaporator: Shanghai Ailang Instrument Co., Ltd., Shanghai, China), and then the residue was dissolved with 2 mL of 75% ethanol. The pre-experiment scheme for extraction of polyphyllin II and polyphyllin VII is shown in Figure 7. All sample solutions were filtered through membrane filters (0.22 μm pore size) before HPLC analysis.

### 3.4. Single Factor Experiment

The fresh leaves of *P. polyphylla* var. *yunnanensis* were ground into powder with liquid nitrogen. A total of 1 g of leaf powder was added to pure water solution at a certain solid-to-liquid ratio (1:5, 1:10, and 1:15 mg/mL) in an ultrasonic cleaner at a certain temperature (30, 35, 40, and 45 °C) for a certain period (15, 30, 60, and 120 min). Then, the powder was extracted in an ultrasonic cleaner with 5 mL of 75% ethanol at 35 °C for 30 min, and the process was repeated three times. The filtered solutions were combined and concentrated to dryness at 50 °C in a rotary evaporator device, and then the residue was dissolved with 2 mL of 75% ethanol. The single factor experiment scheme for extraction of polyphyllin II and polyphyllin VII is shown in Figure 7. All sample solutions were filtered through membrane filters (0.22 μm pore size) prior to the HPLC analysis.

### 3.5. Response Surface Design

Synthesizing the basis of pre-experimentation and one-way experiments, the effects of three factors, namely time (A), temperature (B), and solid-to-liquid ratio (C), on the yields of polyphyllin II and polyphyllin VII were analyzed. Based on the one-way experiment, three levels were determined for each factor. The Design Expert 13.0 software was applied to design 17 groups of 3-factor, 3-level experiments, and the yields of polyphyllin II and polyphyllin VII were the response values (Table 6).

### 3.6. Statistical Analysis and Validation of Data

Statistical software Design Expert 13.0 and SPSS 22.0 were used to statistically analyze the results of the experiments. Each sample in each experiment had three replications. One-way analysis of variance (ANOVA) and Duncan’s multiple range test were used for inter-sample comparisons. The statistical design of the experiments and optimization was carried out by Design Expert software to obtain the regression equations, the significance of each parameter, the response surface plots, and the optimal extraction conditions.

## 4. Conclusions

This study proposes that water-assisted extraction is a more environmentally friendly and cost-effective alternative to enzyme-assisted extraction. Based on the single-factor experiment, the extraction period, extraction temperature, and solid-to-liquid ratio for the water-assisted extraction of polyphyllin II and polyphyllin VII from the leaves of *P. polyphylla* var. *yunnanensis* were determined by response surface design. The optimal extraction conditions for polyphyllin II and polyphyllin VII were as follows: extraction time 57 and 21 min, extraction temperature 36 and 32 °C, and solid-to-liquid ratio 1:10 and 1:5 g/mL, respectively. The yield of polyphyllin II and polyphyllin VII was 1.895 and 5.010% under the conditions. The water-assisted extraction used in this study is economical, efficient, convenient, and has no pollution. This method has practical applications and provides scientific data for the further development of polyphyllin II and polyphyllin VII from the leaves of *P. polyphylla* var. *yunnanensis* in the field of medicine.

## Figures and Tables

**Table 1:** Box–Behnken experimental design and results of polyphyllin II and polyphyllin VII.

Number	A Period (min)	B Temperature (°C)	C Solid-to-Liquid Ratio (g/mL)	Polyphyllin II Yield (%) ^a^	Polyphyllin VII Yield (%) ^a^
1	67.5	35	1:10	1.8547	4.5207
2	15.0	40	1:10	1.8123	4.5329
3	120.0	30	1:10	1.7986	4.3014
4	67.5	30	1:15	1.7855	4.4788
5	67.5	40	1:5	1.8071	4.2982
6	67.5	30	1:5	1.7226	4.9687
7	120.0	35	1:5	1.7434	4.5200
8	67.5	35	1:10	1.8436	4.5766
9	15.0	35	1:5	1.8155	4.8418
10	67.5	35	1:10	1.8614	4.6581
11	67.5	35	1:10	1.8455	4.6141
12	120.0	40	1:10	1.7554	3.7153
13	120.0	35	1:15	1.8198	3.9430
14	15.0	30	1:10	1.7440	4.8699
15	15.0	35	1:15	1.7840	4.6934
16	67.5	40	1:15	1.7656	3.9081
17	67.5	35	1:10	1.8535	4.7187

^a^ Each value is the mean of triplicate measure, *n* = 3.

**Table 2:** Analysis of variance (ANOVA) for the regression equation of polyphyllin II.

Source	Sum of Squares	df	Mean Square	F-Value	*p*-Value
Model	0.0298	9	0.0033	37.41	<0.0001 ***
A	0.0002	1	0.0002	2.11	0.1900
B	0.0010	1	0.0010	11.37	0.0119 *
C	0.0005	1	0.0005	6.21	0.0414 *
AB	0.0031	1	0.0031	35.15	0.0006 **
AC	0.0029	1	0.0029	32.92	0.0007 **
BC	0.0027	1	0.0027	30.82	0.0009 **
A^2^	0.0030	1	0.0030	34.32	0.0006 **
B^2^	0.0094	1	0.0094	106.63	<0.0001 ***
C^2^	0.0049	1	0.0049	55.76	0.0001 **
Residual	0.0006	7	0.0001		
Lack of Fit	0.0004	3	0.0001	2.59	0.1902
Pure Error	0.0002	4	0.0001		
Cor Total	0.0304	16			

* means significant at *p* ≤ 0.05; ** means highly significant at *p* ≤ 0.01; *** means extremely significant at *p* ≤ 0.0001.

**Table 3:** Analysis of variance (ANOVA) for the regression equation of polyphyllin VII.

Source	Sum of Squares	df	Mean Square	F-Value	*p*-Value
Model	1.90	9	0.2115	29.09	<0.0001 ***
A	0.7554	1	0.7554	103.89	<0.0001 ***
B	0.5855	1	0.5855	80.53	<0.0001 ***
C	0.3222	1	0.3222	44.31	0.0003 **
AB	0.0155	1	0.0155	2.13	0.1875
AC	0.0459	1	0.0459	6.32	0.0402 *
BC	0.0025	1	0.0025	0.3425	0.5768
A^2^	0.0329	1	0.0329	4.52	0.0711
B^2^	0.1281	1	0.1281	17.62	0.0040 **
C^2^	0.0037	1	0.0037	0.5128	0.4971
Residual	0.0509	7	0.0073		
Lack of Fit	0.0280	3	0.0093	1.62	0.3178
Pure Error	0.0229	4	0.0057		
Cor Total	1.95	16			

* means significant at *p* ≤ 0.05; ** means highly significant at *p* ≤ 0.01; *** means extremely significant at *p* ≤ 0.0001.

**Table 4:** Results of water-assisted extraction validation tests.

	Number	Yield (%)	Average Yield (%)	RSD (%)
Polyphyllin II	1	1.940		
	2	1.892	1.895	2.300
	3	1.853		
	1	5.083		
Polyphyllin VII	2	4.969	5.010	1.270
	3	4.977		

**Table 5:** HPLC mobile phase procedures.

Time (min)	Water (%)	Acetonitrile (%)
0.0	57.0	43.0
13.0	57.0	43.0
14.0	45.0	55.0
25.0	45.0	55.0

**Table 6:** Experimental factors and level design.

Level		Factor	
	A (min)	B (°C)	C (g/mL)
1	15.0	30	1:5
0	67.5	35	1:10
−1	120.0	40	1:15

## Data Availability

The data presented in this study are available in the article.
